# An epilepsy-associated K_V_3.1 potassium channel variant acts via dominant-positive effect

**DOI:** 10.1085/jgp.202513962

**Published:** 2026-06-25

**Authors:** Jerome Clatot, Hubert Monnerie, Axel Panzer, Konrad Platzer, Ethan M. Goldberg

**Affiliations:** 1 Division of Neurology, Department of Pediatrics, The Children’s Hospital of Philadelphia, Philadelphia, PA, USA; 2 The Epilepsy Neurogenetics Initiative, The Children’s Hospital of Philadelphia, Philadelphia, PA, USA; 3 The Center for Brain Development, Genetics, and Engineering (BRIDGE), The Children’s Hospital of Philadelphia, Philadelphia, PA, USA; 4 DRK Kliniken Berlin Westend, Epilepsy Center/Pediatric Neurology, Berlin, Germany; 5 Institute of Human Genetics, University of Leipzig Medical Center, Leipzig, Germany; 6 Department of Neurology, The University of Pennsylvania Perelman School of Medicine, Philadelphia, PA, USA; 7 Department of Neuroscience, The University of Pennsylvania Perelman School of Medicine, Philadelphia, PA, USA

## Abstract

Variants in *KCNC1* encoding the voltage-gated potassium (K^+^) channel subunit K_V_3.1 are an emerging cause of a spectrum of neurological disease including developmental delay/intellectual disability, ataxia, myoclonus, and epilepsy, including progressive myoclonus epilepsy and developmental and epileptic encephalopathy. Here, we report novel biophysical properties of a recurrent *de novo* missense variant in *KCNC1* c.1196C>T (p.Thr399Met) associated with epilepsy, mild developmental delay, and nonprogressive ataxia. The variant is localized to the highly conserved pore region of the channel, and voltage-clamp electrophysiological recording demonstrated a complete loss of function, as seen in more severe forms of *KCNC1-*related disorders. When expressed with wild-type (WT) to mimic the heterozygous state of the variant as would occur in a disease context, current density was not markedly reduced; however, resulting currents displayed a ∼20 mV hyperpolarizing shift of the voltage dependence of activation along with slowed deactivation kinetics, consistent with gain of function. In order to better understand this “dominant-positive effect” exerted on the WT channel, we co-expressed the K_V_3.1 p.Thr399Met variant with the recurrent p.Ala421Val variant known to act via loss of function with near-complete absence of current and associated with severe *KCNC1-*related disease. Co-expression of the two nonfunctioning variants led to a mild rescue of K^+^ current compared with the *KCNC1-*p.Ala421Val variant alone, further supporting a dominant-positive effect. Both *KCNC1-*p.Thr399Met and p.Ala421Val displayed trafficking deficiency. These results suggest that inclusion of the p.Thr399Met variant in heterotetrameric K_V_3 channels alters the gating kinetics of WT channel subunits, and highlight the unique features of this variant. The apparent complete loss of function of the p.Thr399Met variant when expressed alone is inconsistent with the relatively mild clinical presentation of the patient, subsequently explained via the “dominant-positive” action when combined with WT subunits.

## Teaser

KCNC1 encodes the potassium channel subunit K_V_3.1 critical for high-frequency neuronal firing. Variants cause a spectrum of neurological disorders. While the recurrent p.Thr399Met variant shows complete loss of function alone, co-expression with wild-type subunits reveals a “dominant-positive” gain of function, highlighting complex potassium channel subunit interactions.

## Introduction

Potassium (K^+^) channels are critical determinants of neuronal excitability via setting the resting membrane potential and mediating repolarization of the action potential (Jan and Jan, 2012). Pathogenic variants in various genes encoding K_V_ channel genes are established causes of epilepsy, including *KCNA2*, *KCNB1*, *KCNC1* and *KCNC2*, and *KCNQ2* (Niday and Tzingounis, 2018; Marini et al., 2017; Syrbe et al., 2015; Cooper, 2012). Such findings underscore the central role of K_V_ channels in brain function and the role of K_V_ channel dysfunction in human neurological disease, with implications for targeted therapeutics. K_V_3.1 is one of four members of the K_V_3 subfamily of voltage-gated K^+^ channels (K_V_3.1–3.4), which contribute to high-frequency firing in specific subsets of neurons throughout the nervous system based on unique biophysical properties, including a depolarized voltage dependence of activation, fast activation kinetics, and rapid deactivation, relative to other K_V_ channels (Kaczmarek and Zhang, 2017; Rudy and McBain, 2001). Missense variants in the K_V_3 subfamily member gene *KCNC1* encoding the K^+^ channel subunit K_V_3.1 act via gain of function (GoF) or loss of function (LoF) at the level of the ion channel and are associated with a spectrum of neurological dysfunction: LoF leads to progressive myoclonus epilepsy type 7 (EPM7 or PME7) or developmental and epileptic encephalopathy (DEE) (Cameron et al., 2019; Park et al., 2019; Muona et al., 2015; Oliver et al., 2017; Poirier et al., 2017; Wengert et al., 2026; Ginn and Goldberg, 1993), while GoF is associated with a more mild syndrome including isolated nonprogressive myoclonus and nonspecific developmental delay/intellectual disability (DD/ID), with or without epilepsy that is typically mild (Clatot et al., 2023). However, genotype–phenotype correlation remains incompletely defined.

Here, we describe unique properties of a recurrent *KCNC1* variant that exhibits complete LoF when expressed as a homomer. Two identified patients with this variant present with neurological disease characterized by mild–moderate DD/ID and epilepsy, but not the more severe phenotypes of EPM or DEE previously associated with LoF variants in *KCNC1*. Further investigation revealed the variant to in fact exert a GoF effect via putative “dominant-positive” action when co-expressed with wild-type (WT) subunits. Whole-cell voltage-clamp electrophysiological recordings of K^+^ channels containing *KCNC1-*WT and *KCNC1-*p.Thr399Met subunits in a heterologous mammalian cell line revealed a leftward (hyperpolarized) shift in the voltage dependence of activation and slowed deactivation, which is more consistent with the relatively mild clinical presentation of the case and explains the initial misalignment between clinical phenotype and electrophysiological function of the isolated variant *in vitro*. These mixed biophysical effects are predicted to exert a LoF effect on K_V_3-expressing fast-spiking neurons in the brain (Wengert et al., 2026; Feng et al., 2024).

## Materials and methods

### Plasmid preparation, cell culture, and transfection

A cDNA plasmid encoding human *KCNC1* (reference sequence NM_001112741.2) and variants was synthesized and subcloned into a pCMV plasmid. The human *KCNC2* cDNA (reference sequence NM_139137.4) was synthesized and subcloned into a pCMV-IRES-EGFP plasmid as we described previously (Clatot et al., 2024). For cell-surface expression assays, *KCNC1*-HA was synthesized to include an HA tag (YPYDVPDYA) attached to the first extracellular loop behind T231 as described previously (Xu et al., 2007). HEK293T cells (CRL-3216; ATCC) were grown in 35-mm dishes at 37°C in 5% CO_2_ with DMEM supplemented with 10% heat-inactivated fetal calf serum and 1% penicillin–streptomycin. Cells were transfected with 0.1 μg of pCMV-EGFP and 0.4 μg of either WT h*KCNC1* cDNA, variant, or WT:variant in a 1:1 ratio, using PolyFect transfection reagent (Qiagen) according to the manufacturer’s instructions. 24 hours after transfection, cells were trypsinized and seeded at low density, and single GFP-positive cells were identified for patch-clamp experiments.

### Electrophysiology

Whole-cell patch-clamp biophysical experiments were performed at room temperature using an Axopatch 200B amplifier (Molecular Devices) in extracellular Tyrode’s solution consisting of the following: 150 mM NaCl, 2 mM KCl, 1.5 mM CaCl_2_, 2 mM MgCl_2_, 10 mM HEPES, and 10 mM glucose; pH was adjusted to 7.4 with NaOH. Intracellular solution contained, in mM, the following: 125 KCl, 25 KOH, 1 CaCl_2_, 2 MgCl_2_, 4 Na_2_-ATP, 10 EGTA, and 10 HEPES, with pH adjusted to 7.2 with KOH and osmolarity to 305 mOsm/l with sucrose.

Patch pipettes were fashioned from thin-walled borosilicate glass (Harvard Apparatus) and fire-polished (Zeitz) to a resistance of 1.7–2.5 MΩ in the whole-cell configuration. Voltage errors were reduced via series resistance compensation. Currents were digitized at 30 kHz and filtered at 5 kHz by a low-pass Bessel filter. Data were acquired with pClamp 11 (Axon Instruments). Transient K^+^ currents were measured by performing 100-ms step depolarizations between −85 and +55 mV in increments of 5 mV from a holding potential of −120 mV, and the current–voltage relation was constructed; this was followed by a 100-ms pulse to −40 mV to facilitate measurement of tail current. Activation conductance was normalized, plotted against voltage, and fit with a Boltzmann function to determine *V*_1/2_ of activation. Kinetics of deactivation was calculated via a single exponential fit of the decay of the tail current.

### Cell-surface expression assay

HEK293T cells were cultured and transfected in 35-mm well dishes with 2,000 ng total of *KCNC1* or HA-tagged plasmids. 24 hours after transfection, cells were trypsinized, seeded, and cultured overnight in 24-well plates to obtain a monolayer of 70–80% confluence. DMEM-supplemented serum was removed from each well to be exchanged with plain DMEM containing the mouse monoclonal anti-HA primary antibody (2 µg/μl, Ab49969; Abcam) and incubated for 30 min. Control wells were missing the primary antibody. Live cells were then rinsed three times with PBS to remove the primary antibody, and incubated for another 30 min in plain DMEM containing goat anti-mouse secondary antibody (1:800, LI-COR). After three additional washes to remove the secondary antibody, cells were then fixed in 4% PFA for 20 min, permeabilized with 0.01% Triton solution for 4 min, and then incubated for 1 h with CellTag 700 (1:500, LI-COR). Control wells were incubated without the CellTag 700. Each step was followed by two washes in PBS. Plates were then scanned on an Odyssey LI-COR classic focused at 3.3 µm. The relative cell-surface quantification was achieved by first subtracting the fluorescent background level from wells missing the primary HA antibody and/or CellTag 700. The ratio of fluorescence (Green/Red; 800/700) corresponds to relative cell-surface expression, normalized to the number of cells in each well.

### Data analysis

Data for electrophysiological parameters were obtained from at least *n* = 10 cells randomly selected from *N* = 3 transfections. Data were analyzed using Clampfit 11 (Molecular Devices) and SigmaPlot 15 (Systat Software, Inc.). Results are presented as the mean ± standard error of the mean. Normality was assessed using the Shapiro–Wilk test, and statistical significance was established using one-way ANOVA followed by the Holm–Šidák post hoc correction for multiple comparisons. Statistical significance was set at P < 0.05 with P values reported exactly as provided by SigmaPlot software.

### Online supplemental material

**Figure S1.**
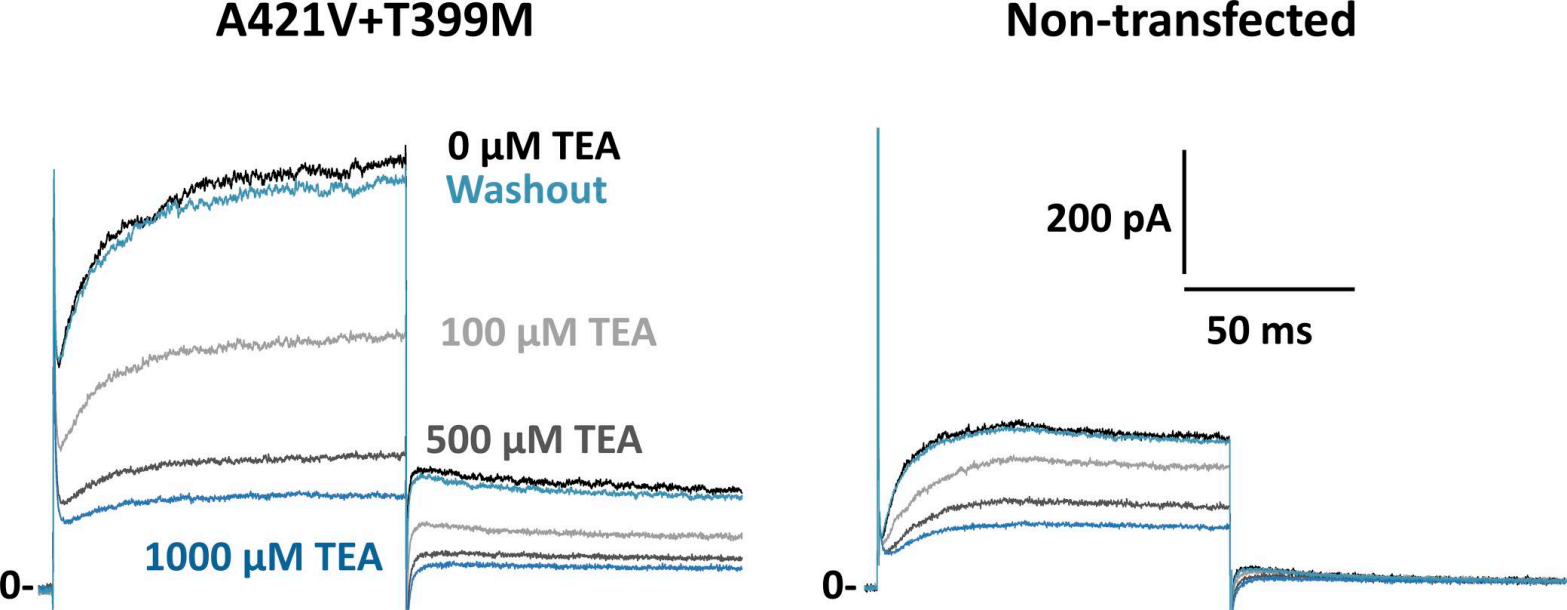
**TEA-sensitive currents in cells expressing K_V_3.1 p.Thr399Met and p.Ala421Val.** Whole-cell recordings were obtained from HEK293T cells co-transfected with K_V_3.1 p.Thr399Met and p.Ala421Val to assess TEA sensitivity. Using the Biopen nanoperfusion system, increasing concentrations of TEA (100 µM → 500 µM → 1 mM) were applied sequentially, followed by washout. In cells expressing the p.Ala421Val + Thr399Met combination, TEA produced a clear, dose-dependent reduction in both the outward K^+^ current and the tail current, confirming that these currents are TEA-sensitive and therefore mediated by K_V_3.1 channels. In contrast, nontransfected cells exhibited no detectable TEA-sensitive currents and lacked tail currents altogether, validating the specificity of the recorded signal.

Fig. S1 depicts variant K^+^ currents sensitive to tetraethylammonium (TEA) ion.

## Results

### Case presentation

Patient 1 is a now 6-year-old boy who presented for medical attention with epilepsy at age 1 year that included myoclonic, myoclonic astatic, and generalized tonic–clonic seizures, which was managed with valproic acid and levetiracetam. Electroencephalography showed generalized discharges of irregular hypersynchronous delta lasting 10 s or less along with generalized and multifocal spike-and-wave complexes. Magnetic resonance imaging (MRI) of the brain was normal. He exhibited mild global developmental delay. He walked at the age 2 years. He could speak in three to four word phrases at age 3 years with relative preservation of receptive language function. Whole-exome sequencing in the index revealed the heterozygous variant in *KCNC1* c.1196C>T (p.Thr399Met). Subsequent Sanger sequencing in the parents confirmed the variant to be of *de novo* origin.

Patient 2 is a 3-year-old boy who developed epilepsy at age 2 years with multiple seizure types including predominantly myoclonic seizures accompanied by atypical absence seizures and rare generalized tonic–clonic seizures. The patient exhibits gait impairment with mild ataxia. Speech/language and cognition are normal. MRI of the brain was normal. Prior EEG was abnormal with paroxysmal slow spike and wave, but the most recent EEG was normal. An epilepsy next-generation sequencing panel (Arnaud et al., 2022) revealed *KCNC1* c.1196C>T (p.Thr399Met).

### Electrophysiological properties of cells expressing p.Thr399Met alone or at the heterozygous state

Prior reports have demonstrated that the recurrent variant *KCNC1-*p.Thr399Met exhibits LoF with near absence of current in *Xenopus* oocytes with a putative dominant-negative action (Park et al., 2019) as seen for other *KCNC1* variants associated with more severe *KCNC1*-related disorders such as EPM7 (due to the recurrent variant *KCNC1-*p.Arg320His) and DEE (due to the recurrent variant *KCNC1-*p.Ala421Val). Yet, previously reported patients and both patients reported here have a relatively mild phenotype with epilepsy well controlled on antiseizure medication and normal development or mild DD/ID. In order to further investigate the genotype/phenotype relationship of the *KCNC1-*p.Thr399Met variant, we expressed WT and the disease-associated *KCNC1* variant in a heterologous mammalian cell system (HEK293T cells) for voltage-clamp electrophysiological recording.

**Figure 1.**
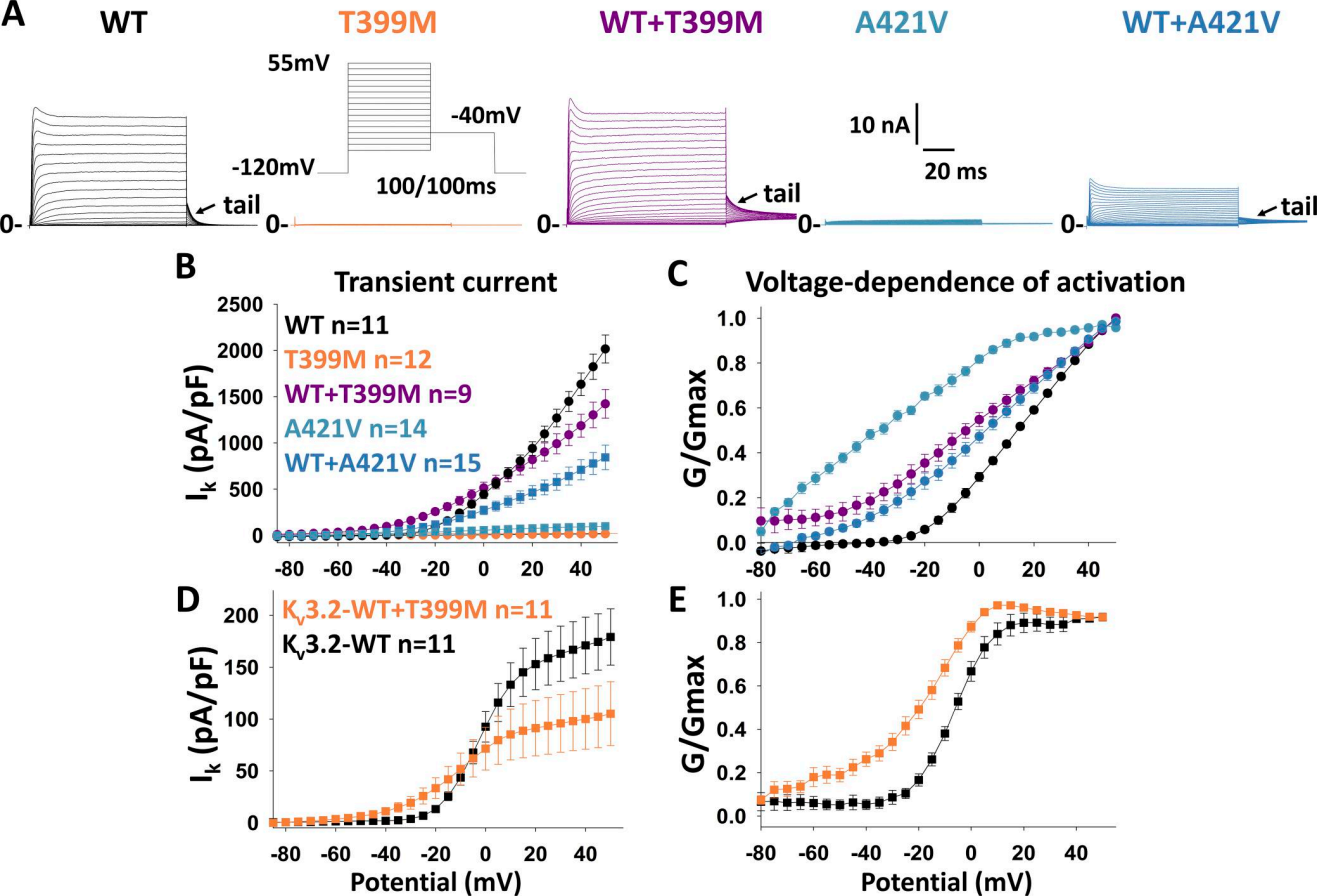
**Biophysical properties of the pathogenic variant *KCNC1-*p.Thr399Met. (A–C)** *KCNC1-*WT (black) or p.Thr399Met variant (T399M; orange) was expressed in HEK293T cells, and recordings were acquired by voltage clamp. **(A)** Representative recordings of K_V_3.1 WT, K_V_3.1 variant Thr399Met, and WT + Thr399Met. **(B)** I-V curves. **(C)** Voltage dependence of activation. **(D)** I-V curves for currents recorded from cells co-expressing K_V_3.1-p.Thr399Met with K_V_3.2-WT. **(E)** Voltage dependence of activation when expressed with K_V_3.2-WT. Note that cells expressing K_V_3.1 or K_V_3.2-WT + K_V_3.1-p.Thr399Met display a prominent left shift of the voltage dependence of activation relative to WT alone.

Noninactivating outward delayed rectifier K^+^ currents were recorded via depolarizing steps from −120 mV (Fig. 1 A). We found a near-complete LoF of the variant *KCNC1-*p.Thr399Met, with 14.7 ± 0.95 pA/pF (*n* = 12) at +40 mV, compared with 1,633.7 ± 122.4 pA/pF (*n* = 11) for the WT expressed alone (P < 0.001; Fig. 1 A). Since K_V_3 channels assemble as tetramers, we then investigated the possibility that p.Thr399Met could exert dominant-negative suppression of the WT subunit-containing channel. To do so, we co-expressed the WT with the p.Thr399Met variant at a 1:1 ratio. However, recorded currents of 1,185.65 ± 124.2 pA/pF (*n* = 9; P = 0.059 relative to WT alone) were inconsistent with a dominant-negative effect. Furthermore, we found a profound −23.4 mV (hyperpolarized) shift of the voltage dependence of activation, consistent instead with GoF (Fig. 1 B). The voltage at half-maximal activation (*V*_1/2_) was 13.7 ± 0.8 mV (*n* = 11) for cells expressing WT and −9.7 ± 6.9 mV (*n* = 9) for cells expressing both WT and p.Thr399Met (P < 0.001) (i.e., a left shift, further consistent with GoF).

We then compared these data with the most severe nonfunctioning variant *KCNC1-*p.Ala421Val. Results showed that the p.Thr399Met alone is even more severe, as the p.Ala421Val variant led to a small but detectable current of 91.4 ± 23.7 pA/pF (*n* = 14; P < 0.001 vs. p.Thr399Met) (Fig. 1, A and B). Cells expressing WT + p.Ala421Val variant displayed a smaller transient outward K^+^ current of 709.5 ± 107.7 pA/pF (*n* = 15) compared with cells expressing WT alone of 1,633.7 ± 122.4 pA/pF (*n* = 11; P < 0.001; Fig. 1, A and B), consistent with LoF.

Rapid deactivation kinetics is hypothesized to be a critical biophysical feature whereby K_V_3.1 facilitates high-frequency firing. Deactivation was assessed via a single exponential fit to the decay of the tail current upon return to −40 mV (Fig. 1, A and C). The time constant (τ) of deactivation was 3.9 ± 0.2 ms (*n* = 11) for *KCNC1*-WT and 10.6 ± 1.2 ms (*n* = 12) for cells expressing both WT and the Thr399Met variant (P = 0.001 vs. WT via one-way ANOVA with post hoc correction for multiple comparisons using the Holm–Šidák test).

Because K_V_3.1 and K_V_3.2 form heteromeric channels in fast-spiking neurons, we next examined whether the K_V_3.1-Thr399Met variant exerts similar effects when incorporated into assemblies with K_V_3.2. Co-expression of K_V_3.1-Thr399Met with K_V_3.2-WT at a 1:1 ratio resulted in a reduction in peak current density from 170.9 ± 27.3 pA/pF for K_V_3.2-WT (*n* = 11) vs. 100.0 ± 29.3 pA/pF for K_V_3.2-WT + Thr399Met (*n* = 11; P = 0.092 vs. WT via one-way ANOVA with post-hoc correction for multiple comparisons using the Holm–Šidák test), which did not reach statistical significance (Fig. 1 D). The heteromeric channels exhibited a pronounced left shift in the voltage dependence of activation of on average ∼18.1 mV (the difference between the mean values of WT and *KCNC1*-p.Thr399Met), demonstrating that Thr399Met confers a gating effect in K_V_3.1/K_V_3.2 heteromultimers similar to that observed for K_V_3.1-WT + K_V_3.1-Thr399Met (Fig. 1 E).

### Cell-surface expression of the *KCNC1*-p.Thr399Met variant

**Figure 2.**
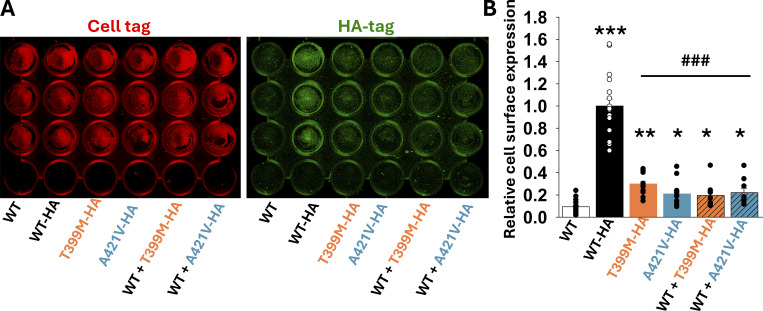
**Cell-surface expression of Thr399Met and Ala421Val. (A)** Representative example of, from left to right, cell-surface expression of HA-tagged K_V_3.1 in HEK293 cells for the following conditions: *KCNC1*-WT (WT), *KCNC1-*WT-HA (WT-HA), *KCNC1-*p.Thr399Met-HA (T399M-HA), p.Ala421Val-HA (A421V-HA), *KCNC1*-WT + p.Thr399Met-HA (WT + T399M-HA), and *KCNC1*-WT + p.Ala421Val-HA (WT + A421V-HA). A cell tag signal (*red*) is proportional to the number of cells in the well, while the HA tag signal (*green*) is proportional to the cell-surface expression of the HA cell-surface tag. **(B)** Quantification of cell-surface expression in A. Note that cells transfected with *KCNC1-*WT (nontagged WT K_V_3.1; WT) display minimal green signal, as expected. Cell-surface expression levels of T399M-HA (*n* = 14; P = 0.002) and A421V variant alone (*n* = 15; P = 0.042) are significantly higher than WT background (*n* = 18), as indicated by asterisks (*). WT + T399M-HA (*n* = 9; P = 0.045) and WT + A421V-HA (*n* = 9; P = 0.047) are also above background, but markedly decreased compared with the cell-surface expression of WT-HA (*n* = 18) as indicated by hatch mark #. *, P < 0.05; **, P < 0.01; *** and ###, P < 0.001. *n* indicates the number of recorded cells per group.

We identified a complete LoF of the *KCNC1*-p.Thr399Met variant, which is localized to the S6 pore domain of the channel. To differentiate between loss of conduction vs. an effect on trafficking, we performed a cell-surface quantification assay after inserting an HA tag on the extracellular loop of the K_V_3.1 channel subunit after Thr231, as described previously (Xu et al., 2007). Results were compared with the *KCNC1-*p.Ala421Val variant. Trafficking of both variants *KCNC1-*p.Thr399Met-HA and p.Ala421Val-HA was markedly decreased to 20.6 ± 0.02% and 19.7 ± 0.03% of the WT-HA (P < 0.001 vs. WT) (Fig. 2). This result suggests that both variants are trafficking-deficient.

To assess whether variants traffic to the cell surface when incorporated into tetramers with WT K_V_3.1, we performed additional cell-surface expression assays in which nontagged WT-K_V_3.1 was co-expressed with HA-tagged Thr399Met or Ala421Val. In both cases, surface abundance of the mutant subunits remained reduced relative to WT-HA alone. However, despite expressing only half as much HA-tagged mutant protein, the surface abundance of Thr399Met-HA and Ala421Val-HA did not decrease further when expressed in a 50:50 ratio with WT (Fig. 2 B). These findings indirectly suggest some level of surface expression of variant K_V_3.1 in the presence of WT subunits. These findings support the electrophysiological observation that a likely small fraction of heterotetramers containing variant subunits is sufficient to produce a pronounced left shift in the voltage dependence of activation.

### The *KCNC1-*p.Thr399Met variant exerts a partial dominant-positive effect on a LoF *KCNC1* variant

**Figure 3.**
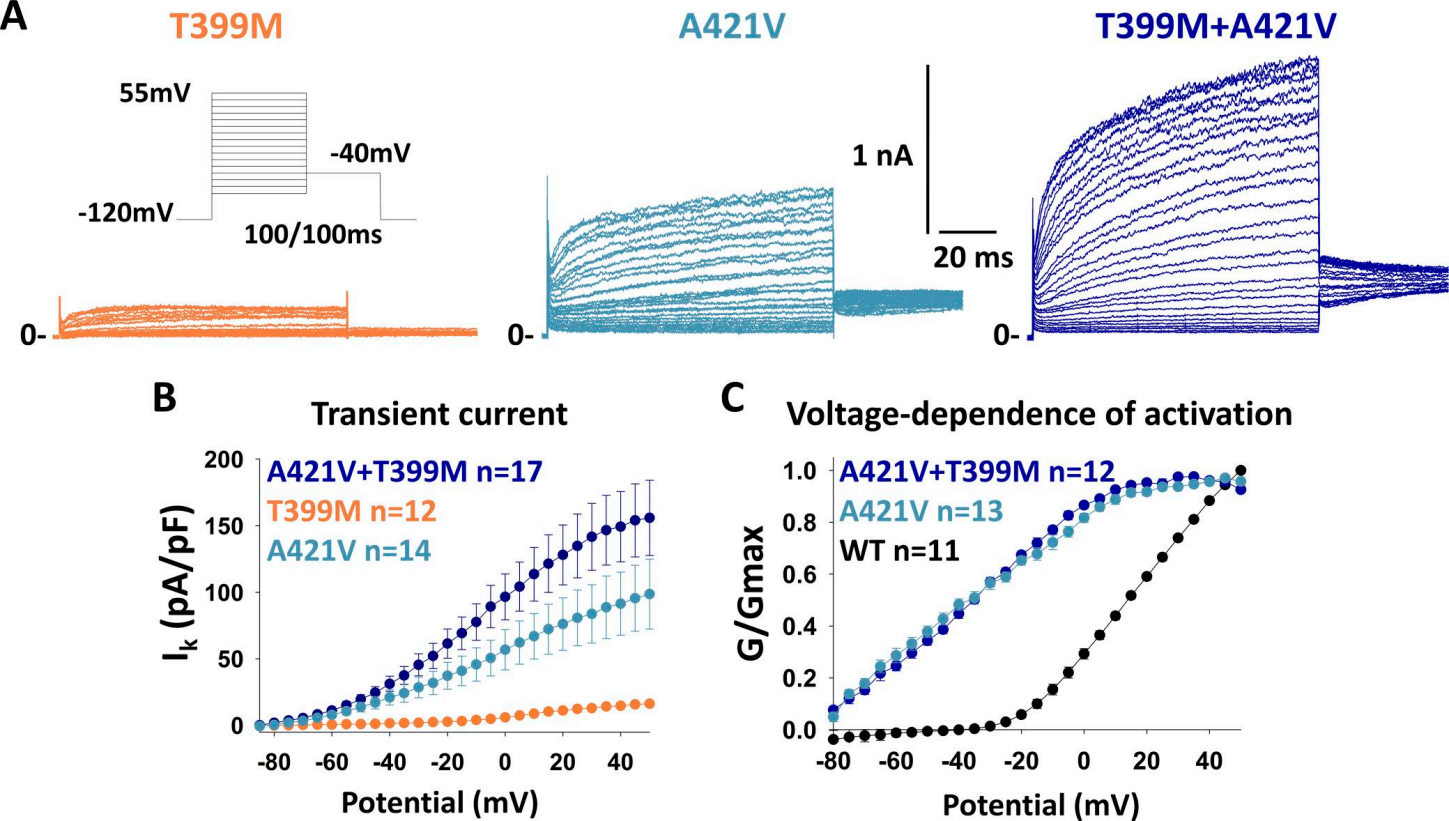
***KCNC1-*p.Thr399Met exerts a dominant-positive effect on the recurrent LoF variant *KCNC1-*p.Ala421Val. (A–C)** WT or p.Thr399Met variant was expressed in HEK293T cells, and recordings were acquired by voltage clamp. **(A)** Representative recordings of *KCNC1-*p.Thr399Met, Ala421Val, and Thr399Met + Ala421Val. **(B)** I-V curves. **(C)** Voltage dependence of activation. Note that cells expressing Thr399Met + Ala421Val show a significantly larger peak current density compared with Ala421Val alone, albeit with no effect on the voltage dependence of activation.

The observation that *KCNC1-*p.Thr399Met exerts a dominant-positive electrophysiological effect on WT subunit–containing K_V_3 channels suggested the possibility that a similar interaction may occur with disease-associated LoF variants and could potentially lead to partial functional improvement. To investigate this, we expressed the *KCNC1-*p.Thr399Met variant with the recurrent LoF variant *KCNC1-*p.Ala421Val associated with DEE. *KCNC1*-p.Thr399Met + p.Ala421Val elicited a significantly larger K^+^ (albeit still quite small) current of 149.3 ± 26.4 pA/pF relative to *KCNC1-*p.Ala421Val expressed alone (Fig. 3, A and B), suggesting that the presence of p.Thr399Met facilitates a mild/partial rescue of p.Ala421Val. Cells transfected with *KCNC1-*p.Ala421Val alone displayed robust TEA-sensitive tail current, whereas nontransfected cells did not; this suggests that the small currents observed in the *KCNC1-*p.Ala421Val–alone condition are indeed K_V_3 currents rather than endogenous and/or “leak” current (Fig. S1). However, we also found a marked ∼49 mV hyperpolarized shift of the mean *V*_1/2_ of activation of cells expressing either p.Ala421Val alone or p.Ala421Val with p.Thr399Met compared with WT. We conclude that the co-expression of the p.Thr399Met variant in combination with p.Ala421Val leads to an increase in transient current consistent with partial rescue, but with no effect on the voltage dependence of activation, which appears to be dominated/defined by the presence of p.Ala421Val.

## Discussion

We present clinical and genetic findings from two previously unreported patients exhibiting normal development or mild global DD/ID, generalized epilepsy responsive to antiseizure medication, and ataxia, but not an EPM/PME or DEE phenotype, found to carry a *de novo* variant p.Thr399Met in the *KCNC1* gene, representing *KCNC1-*related neurological disorder.

*KCNC1* variants give rise to a spectrum of neurological phenotypes, yet the mechanistic basis for this diversity remains incompletely understood (Clatot et al., 2023; Ambrosino et al., 2023). The present study identifies a distinctive behavior of the recurrent *KCNC1-*p.Thr399Met variant that expands the conceptual framework of *KCNC1*-related disorders. Although p.Thr399Met produces complete LoF when expressed as a homomer, its incorporation into heteromeric channels with WT K_V_3.1 (or K_V_3.2) profoundly alters gating, producing a hyperpolarizing shift in activation and slowed deactivation. This seeming paradox of homomeric LoF but heteromeric GoF highlights how subunit-level interactions can generate emergent properties not predictable from homomeric expression alone.

The behavior of *KCNC1-*p.Thr399Met aligns with a growing body of evidence that mixed tetrameric assemblies of WT and variant subunits can exhibit biophysical properties distinct from either component subunit. Recent work on heterozygous BK channel mutations provides a particularly instructive case. Geng et al. demonstrated that the Gly375Arg pathogenic variant in *KCNMA1* generates a set of hybrid tetramers whose activation shifts scale with the number of mutant subunits (from 0 to 4) incorporated into the channel (Geng et al., 2023). The ∼23 mV left shift observed in the macroscopic current recorded from the *KCNC1-*WT + p.Thr399Met co-expression condition is consistent with a model in which even a single mutant subunit biases the activation gate toward opening. This additive–subunit framework offers a compelling explanation for how a homomeric LoF variant can produce a GoF phenotype in the heterozygous state.

Our trafficking data further support this interpretation. Neither *KCNC1-*p.Thr399Met nor p.Ala421Val is fully rescued by WT K_V_3.1; yet, co-expression (in which half as much HA-tagged K_V_3.1-p.Ala421Val is present) did not lead to an apparent decrease in surface abundance despite the mutant being expressed at only half the tagged protein level. This pattern suggests partial heterotetramer formation with mild trafficking rescue insufficient to normalize current density, but adequate to allow a subset of mixed channels to reach the membrane. Because p.Thr399Met strongly stabilizes the open state, even a small population of such channels can exert a disproportionate effect on gating of the macroscopic current, amplifying the functional impact of limited rescue.

An additional layer of complexity arises from the fact that the relative expression of WT and variant alleles in human neurons is unknown. Ion channel genes frequently exhibit unequal allelic expression due to epigenetic regulation, promoter methylation, chromatin accessibility, and stochastic monoallelic expression. Thus, the physiological ratio of WT:variant K_V_3.1 subunits may vary across neuronal populations, potentially amplifying or attenuating the dominant-positive effect observed *in vitro*. Such variability may contribute to the wide phenotypic range associated with *KCNC1* variants.

Together, these findings support a model in which p.Thr399Met acts not simply as a LoF or GoF variant, but as a context-dependent modulator of heteromeric channel behavior. By altering gating in mixed assemblies while remaining nonfunctional as a homomer, p.Thr399Met reveals a previously unrecognized mechanism within the *KCNC1* disease spectrum: a LoF variant that induces GoF through subunit-dependent interactions. This mechanism provides a plausible explanation for the relatively mild clinical presentation associated with p.Thr399Met and highlights the importance of considering heteromeric assembly, trafficking rescue, and allelic imbalance when interpreting the pathogenicity of K_V_3.1 variants.

### Proposed mechanisms of *KCNC1*-related neurological disorders

K_V_3.1 is preferentially found in fast-firing neurons across the brain, including parvalbumin-positive GABAergic inhibitory interneurons (PV-INs) located in the neocortex, hippocampus, amygdala, and basal ganglia. It is also highly expressed in the reticular thalamic nucleus, and various cell types in cerebellum including cerebellar granule cells and deep cerebellar nuclei (Chow et al., 1999; Weiser et al., 1994; Weiser et al., 1995). This expression pattern overlaps with that of K_V_3.2, and in PV-INs, K_V_3 channels are likely composed of heteromeric assemblies of K_V_3.1 and other K_V_3 subunits (mainly K_V_3.2). These subunits share nearly identical electrophysiological characteristics and possess distinctive biophysical traits that support rapid neuronal firing such as a more depolarized activation threshold compared with other K_V_ family members, rapid activation and deactivation kinetics, and little/no inactivation (Kaczmarek and Zhang, 2017; Rudy and McBain, 2001). Previous studies have shown that LoF variants can range in severity and often exert a dominant-negative influence through the tetrameric structure of voltage-gated K^+^ channels. Although the variants discussed here exhibit LoF due to their trafficking deficiency, we also show GoF characteristics, with the combined result being a dominant-positive effect via heterotetramerization with the WT channel.

### Limitations

Geng et al. (2023) used elegant single-channel recordings of BK channels containing the range of possible stoichiometries of WT and disease-associated p.Gly375Arg variants to develop a model in which variant subunits contribute additive effects to channel dysfunction (Geng et al., 2023). We did not perform similar single-channel recordings here, but future work could determine whether a similar incremental/additive model applies to disease-associated variants in *KCNC1* and other epilepsy-associated K^+^ channel genes*. In vivo*, native K_V_3.1-containing K_V_3 channels are likely heteromultimers containing K_V_3.2 and/or K_V_3.3, and hence, the situation is quite complicated.

While the cell-surface expression assay suggests that WT K_V_3.1 does not “rescue” trafficking of variant K_V_3.1-p.Thr399Met, surface expression of K_V_3.1-p.Thr399Met-HA was at similar levels when co-expressed at a 50:50 ratio with WT as compared to K_V_3.1-p.Thr399Met-HA alone, suggesting an incomplete/partial rescue. However, the variant channel is clearly incorporated into at least some channels that successfully traffic to the cell surface, based on the effects on the macroscopic current. The modest impact of K_V_3.1-p.Thr399Met on K_V_3.1-p.Ala421Val suggests the possibility that dominant-positive mechanisms could potentially be further developed therapeutically to rescue disease-associated K^+^ channel variants.

### Conclusion

We have identified a non-EPM/non-DEE subtype of *KCNC1-*related neurological disorder characterized by generalized epilepsy and normal development or mild global developmental delay along with mild nonprogressive ataxia resulting from the recurrent *de novo* heterozygous variant *KCNC1*-p.Thr399Met. We propose a dominant-positive action to explain the apparent disconnect between a relatively mild clinical presentation along the spectrum of *KCNC1*-related neurological disorders and the marked LoF at the channel level when the p.Thr399Met variant is expressed alone. The precise neuronal and circuit-level mechanisms by which *KCNC1* variants cause disease remain to be fully elucidated, as does the basis for the diverse clinical manifestations seen across *KCNC1*-related disorders.

## Data Availability

Raw data are available upon request.
